# A Systematic Review and Meta-analysis of the Prevalence of Community-Onset Bloodstream Infections among Hospitalized Patients in Africa and Asia

**DOI:** 10.1128/AAC.01974-19

**Published:** 2019-12-20

**Authors:** Christian S. Marchello, Ariella P. Dale, Sruti Pisharody, Matthew P. Rubach, John A. Crump

**Affiliations:** a Centre for International Health, University of Otago, Dunedin, New Zealand; b Colorado Department of Public Health and Environment, Denver, Colorado, USA; c Duke University School of Medicine, Durham, North Carolina, USA; d Division of Infectious Diseases and International Health, Department of Medicine, Duke University Medical Center, Durham, North Carolina, USA

**Keywords:** antimicrobial resistance, bacteremia, bloodstream infections, community-onset infections

## Abstract

Community-onset bloodstream infections (CO-BSI) are major causes of severe febrile illness and death worldwide. In light of new data and the growing problem of antimicrobial resistance (AMR) among pathogens causing BSI, we undertook a systematic review of hospital-based studies of CO-BSI among patients hospitalized with fever. Without restriction to language or country, we searched PubMed, Web of Science, and Scopus for prospective hospital-based studies of culture-confirmed CO-BSI among febrile inpatients. We determined by study the prevalence of BSI among participants, the pathogens responsible for BSI, and the antimicrobial susceptibility patterns of pathogens causing BSI, according to place and time. Thirty-four (77.3%) of 44 eligible studies recruited 29,022 participants in Africa and Asia combined. Among participants in these two regions, the median prevalence of BSI was 12.5% (range, 2.0 to 48.4%); of 3,220 pathogens isolated, 1,119 (34.8%) were Salmonella enterica, 425 (13.2%) Streptococcus pneumoniae, and 282 (8.8%) Escherichia coli. Antimicrobial susceptibility testing was reported in 16 (36.4%) studies. When isolates collected prior to 2008 were compared to those collected in the period of 2008 through 2018, the proportions of typhoidal *Salmonella* and Staphylococcus aureus isolates resistant to several clinically relevant antimicrobials increased over time, while S. pneumoniae susceptibility was stable. CO-BSI remain a major cause of severe febrile illness among hospitalized patients in Africa and Asia, with S. enterica, S. pneumoniae, and E. coli predominating. There is a concerning increase in AMR among serious infections caused by community-onset pathogens. Ongoing surveillance is needed to inform empirical management and strategies to control AMR.

## Abstract (precis)

Community-onset bloodstream infections (CO-BSI) are major causes of severe febrile illness and death worldwide. In light of new data and the growing problem of antimicrobial resistance (AMR) among pathogens causing BSI, we undertook a systematic review of hospital-based studies of CO-BSI among patients hospitalized with fever.

## INTRODUCTION

Fever is a common reason for persons to seek health care at an emergency department (ED) or inpatient facility (1–4). While febrile illnesses seen at the community or primary care level are often self-limiting (5, 6), patients with severe illness commonly are among those presenting for hospital care (2, 7–11). With declines in malaria as a cause of febrile illness in low-resource areas (12, 13), attention to other causes of severe febrile illness, including bloodstream infections (BSI), has increased (14). Timely administration of appropriate empirical antimicrobial therapy can be life-saving, but designing the most appropriate empirical antimicrobial regimen requires a robust understanding of common causes of bacteremia and their patterns of antimicrobial resistance (AMR).

The patterns of organisms causing hospital-acquired and health care-associated BSI differ from those causing community-onset BSI (CO-BSI) (15). Hospital-acquired infections are defined as those with onset >48 h after hospital admission (16), and health care-associated infections are those associated with recent hospital admission or exposure to health care facilities (17, 18). AMR has been increasing among some pathogens in the community (19–21), risking mismatches between empirical antimicrobial regimens and etiological agents. For low-resource settings, standardized, high-quality laboratory services to identify AMR may be limited and local or national surveillance data may be unavailable (22, 23). In this context, sentinel site studies often provide the best evidence to inform management and to monitor trends in AMR (24).

Two systematic reviews described the epidemiology of CO-BSI in Africa and Asia, through 2009 and 2010, respectively (25, 26). Since then, new data have been published and concern has grown regarding AMR among community-onset pathogens, necessitating changes in international guidelines for empirical therapy of severe febrile illness (27, 28).

We performed a systematic review and meta-analysis to update and to expand previous reviews, to inform the empirical management of severe febrile illness, and control strategies for major pathogens. Our review was designed to inform the management of BSI in patients presenting with severe febrile illness, rather than BSI in patients meeting the definition of sepsis, which is a distinct clinical problem that been reviewed by others (29, 30).

(This work was presented in part at ASM Microbe 2019, San Francisco, CA, 20 to 24 June 2019.)

## RESULTS

**FIG 1:**
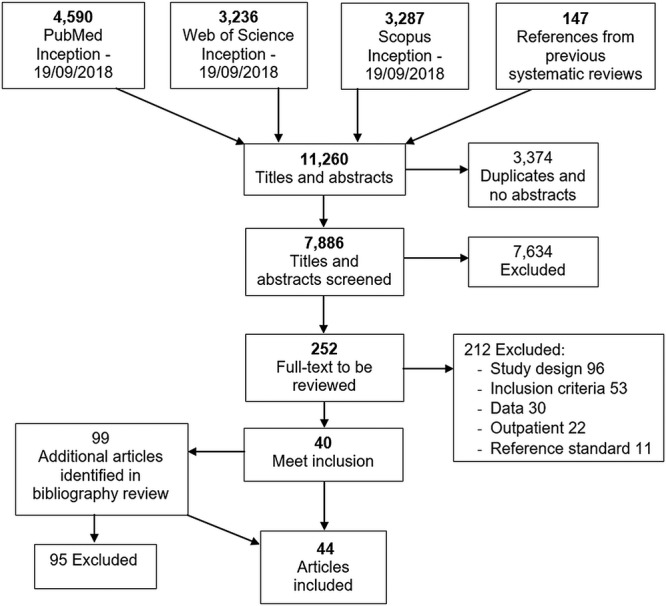
Flow diagram of search strategy and selection of articles reporting the prevalence of CO-BSI among febrile hospitalized patients in 1946 through 2018.

Our search of three online databases yielded 11,113 articles (Fig. 1). After the addition of 147 references from the two prior systematic reviews and the removal of 3,374 duplicates, a total of 7,886 titles and abstracts were screened for inclusion. We excluded 7,634 abstracts, leaving 252 full-text articles to screen. We excluded 190 articles based on study design, improper inclusion criteria, insufficient data to abstract, or inadequate reference standard diagnostics. Another 22 articles describing outpatient studies were excluded. Screening of the bibliographies of the included articles added 4 eligible articles, resulting in 44 articles included for analysis (31–74). Quality assessment is available in Table S1 in the supplemental material.

### Study characteristics.

**FIG 2:**
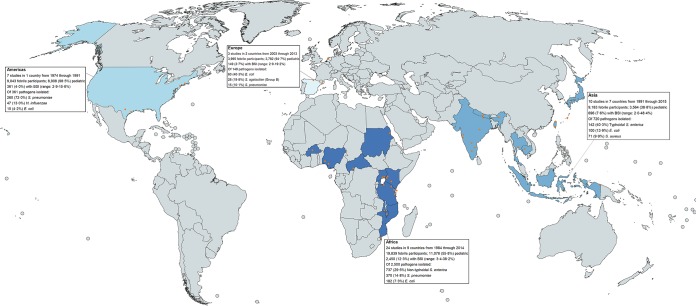
World map of hospital-based study locations and summary findings on prevalent pathogens causing CO-BSI among febrile hospitalized patients in 1946 through 2018 (created using MapChart).

The 44 studies collected data from 1974 through 2015 in 19 countries, recruiting 42,060 participants, of whom 3,656 (8.7%) had BSI (Table 1). Eighteen studies were published from 2010 through 2018; among those, 1,418 (7.0%) of 20,399 participants had BSI, compared to 2,238 (10.3%) of 21,661 participants in studies published prior to 2010. According to United Nations geographical subregion, Eastern Africa had 19 studies performed in 5 countries, the most of any subregion (Fig. 2). All studies in Northern America collected data before 1991 and were performed in the United States (39, 47, 55, 57, 58, 70, 74). Two studies were performed in Southern Europe (42, 59) and 1 in Western Europe (53); no studies from the Southern Africa, Central and West Asia, Eastern and Northern Europe, Latin America and the Caribbean, or Oceania subregions were identified. Blood culture contamination prevalence was reported in 24 (54.5%) studies, and 3 (6.8%) studies reported blood culture volume adequacy (37, 40, 41).

**TABLE 1:** Characteristics of 44 included studies of global CO-BSI among febrile hospitalized patients, according to United Nations geographic region and subregion classification, collecting data 1974 through 2015

Region and subregion	Locality and country	Data collection period	Inclusion age (median)^*a*^	Fever criterion	Recruitment setting	No. of febrile patients	No. of hospitalized patients with BSI
Africa							
Eastern Africa	West Kenya, Kenya (66)	1987–1990	>8 yr (NR)	>38°C	2 regional hospitals	449	58
	Mumias, Kenya (43)	1994	>5 yr (NR)	≥38°C	Private regional hospital	229	51
	Nairobi, Kenya (63)	2001	3 mo to 12 yr (mean, 32 mo)	>37.5°C	University teaching hospital	264	32
	Multiple, Kenya (64)	2013–2014	6 mo to 5 yr (3.1 yr)	≥37.5°C	1 teaching and referral hospital and 2 district hospitals	148	5
	Blantyre, Malawi (73)	1996–1997	Children (NR)	≥38°C	Pediatric wards of 1,100-bed teaching hospital	2,123	NR (365 isolates)
	Blantyre, Malawi (44)	1997–1998	Adults (NR)	>37.5°C	Medical ward of large government teaching hospital	2,789	449
	Lilongwe, Malawi (35)	1998	≥14 yr (29 yr)	≥37.5°C	Medical service of 300-bed medical center	238	67
	Blantyre, Malawi (65)	2000	≥14 yr (NR)	≥37.4°C or shock or history of fever in past 4 days	Medical wards of large government hospital	352	128
	Maputo, Mozambique (67)	2011–2012	≥18 yr (NR)	≥38°C	Internal medicine ward of national referral hospital	841	63
	Dar es Salaam, Tanzania (32)	1995	≥15 yr (38 yr)	≥37.5°C	Adult medical unit of >1,000-bed hospital	517	145
	Dar es Salaam, Tanzania (36)	2001–2002	0–7 yr (8.5 mo)	≥38°C	>1,000-bed hospital	1,787	127
	Muheza, Tanzania (61)	2006–2007	2 mo to 13 yr (1.6 yr)	Current fever or history of fever in past 48 h	District hospital	3,639	341
	Moshi, Tanzania (40)	2007–2008	≥13 yr (38 yr)	≥38°C	2 regional hospitals	403	68
	Moshi, Tanzania (41)	2007–2008	≥2 mo to <13 yr (2 yr)	History of fever in past 48 h or ≥37.5°C	Pediatric ward in large consultant hospital	467	16
	Muheza, Tanzania (62)	2007	≥13 yr (36.5 yr)	Fever or history of fever	District hospital	198	26
	Pemba Island, Tanzania (71)	2009–2010	>2 mo (NR)	≥37.5°C	3 district hospitals	2,209	79
	Mwanza, Tanzania (38)	2011–2012	2–60 mo (18 mo)	≥37.5°C at time of admission	Pediatric ward of medical center	317	21
	Kampala, Uganda (69)	1997	15–65 yr (30 yr)	>38°C	Medical wards of large public teaching hospital	305	72
	Jinja, Uganda (50)	2012	6 to <60 mo (15.5 mo)	<37.5°C or history fever in past 24 h	ED of regional referral hospital	250	45
Middle Africa	Bangui, Central African Republic (48)	1999	All ages (32 yr)	None given	Department of medicine of 44-bed reference community hospital	131	46
Northern Africa	Port Sudan, Sudan (46)	1984	≥12 yr (mean, 29 yr)	≥37.8°C	Regional hospital	100	22
Western Africa	Benin City, Nigeria (31)	1988–1989	1 mo to 5 yr (NR)	≥38°C	Pediatric ED at university hospital	642	67
	Ibadan, Nigeria (34)	1998	1–12 mo (4.6 mo for those with septicemia)	≥38°C	Pediatric ED at university hospital	102	39
	Boulkiemde, Burkina Faso (45)	2013–2014	2 mo to 15 yr (24.6 mo)	≥37.5°C or history of fever in past 48 h	Pediatric ward of referral hospital and healthcare center	1,339	118
Asia							
East Asia	Tainan, Taiwan (51)	2006–2007	≥18 yr (mean, 53.8 yr)	>38°C for <1 wk	ED of area medical center	396	60
	Okinawa, Japan (72)	NR	≥15 yr (mean, 57 yr)	≥38°C	Large community hospital serving 400,000	526	40
	Taipei, Taiwan (54)	NR	≤15 yr (NR)	≥39°C	Emergency services of hospital	300	6
South-eastern Asia	Bangkok, Thailand (33)	1997	≥15 yr (32 yr)	≥38°C	Medical service of 500-bed hospital	246	119
	Multiple, Thailand (52)	1991–1993	>2 yr (NR)	>38.3°C for 3–14 days	10 community-based hospitals	1,137	36
	Jayapura, Northeastern Papua, Indonesia (68)	1997–2000	All ages (25 yr)	History of fever or ≥38°C at admission	Provincial hospital serving 286,000	226	34
	Siem Reap, Cambodia (37)	2009–2010	<16 yr (2.0 yr)	≥38°C within 48 h after admission	50-bed children's hospital	1,225	76
South Asia	Kathmandu, Nepal (49)	2005–2006	≤12 yr (NR)	>38.3°C or afebrile with possible meningitis, pneumonia, or septicemia	Pediatric ward of large referral hospital	2,039	142
	Multiple, India (60)	2011–2012	≥5 yr (31 yr)	≥38°C for 2–14 days	8 secondary community (100–500-bed) hospitals	1,564	124
	Pune, India (56)	2013–2015	>6 mo (29 yr for adults; 2 yr in children)	≥38°C for ≥24 h	Inpatient medicine and pediatric wards of large tertiary public teaching hospital	1,524	59
Europe							
Southern Europe	Bilbao and Barcelona, Spain (59)	2003–2008	<3 mo (NR)	≥38°C	EDs of 2 tertiary teaching hospitals	381	8
	Multiple, Spain (42)	2011–2013	<91 days (NR)	≥38°C	19 EDs	3,401	100
Western Europe	Amsterdam, Netherlands (53)	2008–2009	Adults (66 yr)	>38.2°C	ED of general teaching hospital	213	NR (41 isolates)
Americas							
Northern America	New Haven, Connecticut, USA (57)	1974–1975	<24 mo (NR)	≥40°C	Pediatric ED of large area hospital	330	24
	Texas, USA (39)	1982–1984	6 mo to 2 yr (NR)	≥39.4°C	EDs of 2 community hospitals	201	21
	Philadelphia, Pennsylvania, and Chicago, Illinois, USA (47)	1982–1984	3–36 mo (mean, 16.7 mo)	≥39°C	EDs of 2 children's hospitals	955	42
	Houston, Texas, USA (55)	1983	<24 mo (NR)	Acute febrile illness	ED of children's hospital	570	44
	New Haven, Connecticut, USA (58)	1982–1983	≥16 yr (NR)	≥37.9°C	Internal medicine department of ED at large hospital	135	21
	Chicago, Illinois, USA (74)	1983–1984	3–24 mo (mean, 12.5 mo)	≥40°C	EDs of 2 hospitals	233	17
	Multiple, USA (70)	1987–1991	90 days to 36 mo (12.4 mo)	≥39°C	EDs of 10 hospitals	6,619	192
Total^*b*^						42,060	3,656^*b*^

a NR, number of hospitalized patients with bloodstream infection not reported; number of isolates provided in parentheses as assumed equivalent.

b Includes isolates from Limper et al. (53) and Walsh et al. (73).

### Prevalence of BSI in Africa and Asia.

Thirty-four studies recruited 29,022 participants in Africa and Asia combined, of whom 28,588 (98.5%) had an aerobic blood culture (Table 2). Among participants, 3,146 (10.8%) had BSI, and the median prevalence of BSI was 12.5% (range, 2.0 to 48.4%). There were 3,220 pathogenic organisms isolated among study participants with BSI. Of 3,220 pathogens, 1,996 (62.0%) were Gram-negative bacteria, 854 (26.5%) were Gram-positive bacteria, 94 (2.9%) were yeasts, and 276 (8.6%) were other pathogenic organisms.

**TABLE 2:** Organisms isolated from blood cultures among febrile hospitalized patients in 34 studies in Africa and Asia in 1984 through 2018

Organism group and species isolated	No. of isolates (% of total isolates)	No. of isolates from adults (% of isolates from adults)	No. of isolates from children (% of isolates from children)
*Enterobacteriaceae*	1,676 (52.0)	861 (48.2)	815 (56.9)
Salmonella enterica	1,119 (34.8)	558 (31.2)	561 (39.1)
Typhoidal *Salmonella*^*a*^	328 (10.2)	195 (10.9)	133 (9.3)
S. enterica serovar Typhi	273 (8.5)	146 (8.2)	127 (8.9)
S. enterica serovar Paratyphi A	11 (0.3)	5 (0.3)	6 (0.4)
Nontyphoidal *Salmonella*	758 (23.5)	333 (18.6)	425 (29.7)
S. enterica serovar Typhimurium	399 (12.4)	221 (12.4)	178 (12.4)
S. enterica serovar Enteritidis	126 (3.9)	73 (4.1)	53 (3.7)
Other S. enterica serovars^*b*^	7 (0.2)	4 (0.2)	3 (0.2)
No serovar presented	226 (7.0)	35 (2.0)	191 (13.5)
Unspecified Salmonella enterica	33 (1.0)	30 (1.7)	3 (0.2)
Non-Salmonella enterica *Enterobacteriaceae*	557 (17.3)	303 (17.0)	254 (17.7)
Escherichia coli	282 (8.8)	189 (10.6)	93 (6.5)
*Enterobacter* spp.	105 (3.3)	20 (1.1)	85 (5.9)
*Klebsiella* spp.	91 (2.8)	55 (3.1)	36 (2.5)
*Proteus* spp.	12 (0.4)	9 (0.5)	3 (0.2)
Proteus mirabilis	6 (0.2)	6 (0.3)	0 (0.0)
*Citrobacter* spp.	16 (0.5)	3 (0.2)	13 (0.9)
*Shigella* spp.	8 (0.2)	7 (0.4)	1 (0.1)
Morganella morganii	5 (0.2)	5 (0.3)	0 (0.0)
Other *Enterobacteriaceae*^*c*^	38 (1.2)	15 (0.8)	23 (1.6)
Other Gram-negative organisms	320 (9.9)	122 (6.8)	198 (13.8)
Haemophilus influenzae	78 (2.4)	4 (0.2)	74 (5.2)
Haemophilus influenzae type b	46 (1.4)	4 (0.2)	42 (2.9)
Acinetobacter spp.	55 (1.7)	36 (2.0)	19 (1.3)
Acinetobacter baumannii	8 (0.2)	3 (0.2)	5 (0.3)
*Pseudomonas* spp.	54 (1.7)	21 (1.2)	33 (2.3)
Pseudomonas aeruginosa	22 (0.7)	4 (0.2)	18 (1.3)
*Neisseria* spp.	28 (0.9)	24 (1.3)	4 (0.3)
*Neisseria meningitides*	21 (0.7)	17 (1.0)	14 (1.0)
*Alcaligenes* spp.	11 (0.3)	1 (0.1)	10 (0.7)
Burkholderia pseudomallei	7 (0.2)	1 (0.1)	6 (0.4)
Burkholderia cepacia	6 (0.2)	6 (0.4)	0 (0.0)
Sphingomonas paucimobilis	6 (0.2)	4 (0.2)	2 (0.1)
Additional Gram-negative organisms^*d*^	20 (0.6)	13 (0.7)	7 (0.5)
Unspecified Gram-negative organisms	55 (1.7)	12 (0.7)	43 (3.0)
Gram-positive organisms	854 (26.5)	450 (25.2)	404 (28.2)
Streptococcus pneumoniae	425 (13.2)	248 (13.9)	177 (12.4)
Staphylococcus aureus	241 (7.5)	113 (6.3)	128 (8.9)
*Enterococcus* spp.	56 (1.7)	18 (1.0)	38 (2.7)
Streptococcus agalactiae (group B)	16 (0.5)	1 (0.1)	15 (1.0)
Streptococcus pyogenes (group A)	29 (0.9)	14 (0.8)	15 (1.0)
*Streptococcus* group D	13 (0.4)	2 (0.1)	11 (0.8)
*Streptococcus* group G	2 (0.1)	2 (0.1)	0 (0.0)
Other *Streptococcus* spp.^*e*^	42 (1.4)	31 (1.7)	11 (0.8)
Other Gram-positive organisms^*f*^	11 (0.3)	10 (0.6)	1 (0.1)
Unspecified Gram-positive organisms	19 (0.6)	11 (0.6)	8 (0.6)
Yeasts	94 (2.9)	78 (4.4)	16 (1.1)
Cryptococcus neoformans	61 (1.9)	61 (3.4)	0 (0.0)
Other *Cryptococcus* spp.^*k*^	3 (0.2)	3 (0.1)	0 (0.0)
*Candida* spp.	20 (0.6)	5 (0.3)	15 (1.0)
Histoplasma capsulatum	5 (0.2)	5 (0.3)	0 (0.0)
*Talaromyces marneffei*	4 (0.1)	4 (0.2)	0 (0.0)
Unspecified yeast	1 (<0.1)	0 (0.0)	1 (0.1)
Mycobacteria^*g*^	245 (7.6)	245 (13.3)	0 (0.0)
Mycobacterium tuberculosis complex	206 (6.4)	206 (11.1)	0 (0.0)
Mycobacterium avium complex	28 (0.9)	28 (1.5)	0 (0.0)
Mycobacterium simiae	4 (0.1)	4 (0.2)	0 (0.0)
Other *Mycobacterium* spp.^*h*^	3 (0.1)	3 (0.2)	0 (0.0)
Unspecified *Mycobacterium*	4 (0.1)	4 (0.2)	0 (0.0)
Other unspecified or unidentified organisms	31 (1.0)	31 (1.7)	0 (0.0)
Organisms isolated	3,220^*i*^	1,787	1,433
BSI^*j*^	3,146 (10.8)	1,746 (12.1)	1,400 (9.6)
Febrile inpatients	29,022	14,380	14,642

a Forty-four isolates were classified as serovar Typhi/Paratyphi by Morch et al. (60).

b Including serovars Choleraesuis (*n* = 3), Newport (*n* = 1), Brancaster (*n* = 1), Freetown (*n* = 1), and Infantis (*n* = 1).

c Including coliforms (*n* = 17), *Klebsiella*/*Enterobacter* unspecified (*n* = 15), *Pantoea* spp. (*n* = 3), *Plesiomonas* spp. (*n* = 2), and *Providencia* sp. (*n* = 1).

d Including *Serratia* spp. (*n* = 5), *Aeromonas* spp. (*n* = 4), *Campylobacter* spp. (*n* = 2), *Bacteroides* spp. (*n* = 2), Moraxella catarrhalis (*n* = 1), *Pasteurella* sp. (*n* = 1), Xanthomonas maltophilia (*n* = 1), CDC group 3 (*n* = 1), Vibrio cholerae (*n* = 1), Stenotrophomonas maltophilia (*n* = 1), and *Flavobacterium* sp. (*n* = 1).

e Including *Streptococcus viridans* (*n* = 3) when the study classified it as BSI, although it was likely a contaminant.

f Including *Aerococcus* spp. (*n* = 5), Rhodococcus equi (*n* = 4), *Nocardia* sp. (*n* = 1), and Clostridium perfringens (*n* = 1).

g Only 2,115 of 42,060 participants received mycobacterial blood cultures.

h Including Mycobacterium scrofulaceum (*n* = 2) and Mycobacterium sherrisii (*n* = 1).

i The number isolated is greater than the number of BSI due to polymicrobial infections.

j Values in parentheses indicate proportions of febrile inpatients.

k Including Cryptococcus laurentii (*n* = 2) and unspecified *Cryptococcus* spp. (*n* = 1).

Salmonella enterica accounted for 1,119 (34.8%) of 3,220 pathogens isolated, followed by 425 (13.2%) Streptococcus pneumoniae isolates and 282 (8.8%) Escherichia coli isolates. Of 1,119 S. enterica isolates, 328 (29.3%) were typhoidal *Salmonella* and 758 (67.7%) were nontyphoidal *Salmonella* (NTS). Of typhoidal *Salmonella* isolates, 273 (83.2%) were S. enterica serovar Typhi, 11 (3.4%) were S. enterica serovar Paratyphi A, and 44 (13.4%) were untyped typhoidal *Salmonella*. Of NTS isolates, 399 (52.6%) were S. enterica serovar Typhimurium and 126 (16.6%) were S. enterica serovar Enteritidis.

Of 29,022 febrile participants in Africa and Asia, 14,642 (50.5%) were from pediatric studies and 14,380 (49.5%) were from adult studies. BSI were identified in 1,400 (9.6%) children and 1,746 (12.1%) adult participants (χ^2^ = 49.7; *P* < 0.001). Of the 1,433 and 1,787 pathogens isolated in pediatric and adult studies, respectively, Haemophilus influenzae type b accounted for 42 (2.9%) from pediatric studies and 4 (0.2%) from adult studies (χ^2^ = 39.5; *P* < 0.001), S. pneumoniae for 177 (12.4%) from pediatric studies and 248 (13.9%) from adult studies (χ^2^ = 1.5; *P* = 0.223), and E. coli for 93 (6.5%) from pediatric studies and 189 (10.6%) from adult studies (χ^2^ = 16.1; *P* < 0.001). Mycobacteria were isolated exclusively in adult studies, and no mycobacteria were isolated in the 2 pediatric studies that used mycobacterial blood culture (36, 64).

### Mycobacteria and malaria.

Of 9 studies with 4,036 participants reported using mycobacterial blood culture (32, 33, 35, 36, 40, 48, 64, 65, 69), 8 (88.9%) were performed in Africa and 1 (11.1%) in Asia (33). Among 4,036 participants for whom mycobacterial blood cultures were collected, 245 (6.1%) mycobacterial isolates were recovered. Of mycobacterial isolates, 206 (84.1%) belonged to Mycobacterium tuberculosis complex, 28 (11.4%) belonged to Mycobacterium avium complex, and 11 (4.5%) were other mycobacteria.

Fifteen studies (34.1%) used blood film microscopy to test 10,451 participants for malaria and identified parasitemia in 4,301 (41.2%) (31, 32, 35, 36, 38, 40, 41, 43, 45, 46, 60–65). Eight studies reported malaria and BSI coinfection (31, 32, 43, 45, 61–63, 65). Of 7,168 participants, 3,714 (51.8%) had malaria parasitemia. Of 3,714 participants with malaria parasitemia, 198 (5.3%) also had BSI; among the 3,454 participants with no malaria detected, 710 (20.6%) had BSI (odds ratio [OR], 0.22 [95% confidence interval [CI], 0.18 to 0.26]). Two studies reported parasitemia with specific BSI pathogens (62, 65). Twelve (27.3%) studies with 8,109 participants tested patients for HIV using either serological or nucleic acid amplification methods and described BSI coinfection (32, 33, 35, 36, 40, 41, 48, 61, 62, 65, 67, 69). Among the 2,513 HIV-infected participants, 676 (26.9%) had BSI; 566 (10.1%) of 5,596 HIV-uninfected participants had BSI (OR, 3.2 [95% CI, 2.8 to 3.7]). Associations of HIV with specific pathogens, such as M. tuberculosis and NTS, are provided in Table S2 in the supplemental material.

### BSI prevalence by region.

When stratified by region, the median prevalence of BSI was 14.6% (range, 3.4 to 38.2%) in Africa, 7.3% (range, 2.0 to 48.4%) in Asia, 2.9% (range, 2.1 to 19.2%) in Europe, and 7.3% (range, 2.9 to 15.6%) in the Americas. Of the 2,500 pathogens isolated in Africa, 737 (29.5%) were NTS, followed by 370 (14.8%) S. pneumoniae and 182 (7.3%) E. coli. Of the 720 pathogens isolated in Asia, 142 (19.7%) were typhoidal *Salmonella*, 100 (13.9%) were E. coli, 71 (9.9%) were Staphylococcus aureus, and 55 (7.6%) were S. pneumoniae. Nine NTS pathogens (1.3%) were isolated in Asia, all from a single study (33).

Ten studies were performed in the Americas and Europe regions, of which 2 were adult studies (53, 58). In a multicenter study of 3,401 participants <3 months of age in Spain in 2011 through 2013, E. coli accounted for 46 (46.0%) of 100 pathogens isolated (42). S. pneumoniae represented 260 (72.0%) of the 361 pathogens isolated in 7 studies performed in the United States, followed by 47 H. influenzae pathogens (13.0%), of which 19 (40.0%) were specified as type b.

### Antimicrobial susceptibility.

The results of antimicrobial susceptibility testing were reported in 16 studies (36.4%), all located in Eastern Africa and South and South-eastern Asia (Table 3) (32, 33, 35–38, 40, 41, 43, 44, 49, 61, 67, 68, 71, 73). Eight studies reported antimicrobial susceptibility results for typhoidal *Salmonella* (35, 37, 43, 44, 49, 68, 71, 73). The proportions of typhoidal *Salmonella* isolates susceptible to ampicillin, chloramphenicol, and trimethoprim-sulfamethoxazole for the earlier period versus the contemporary period were 99 (100%) of 99 isolates versus 22 (48.9%) of 45 isolates, 125 (99.2%) of 126 isolates versus 23 (51.1%) of 45 isolates, and 52 (81.3%) of 64 isolates versus 23 (51.1%) of 45 isolates, respectively.

**TABLE 3:** Earlier versus contemporary antimicrobial susceptibilities of prevalent BSI isolates in Africa and Asia in 1994 to 2018

Organism, group,^*a*^ and antimicrobial	Earlier period (pre-2008)			Contemporary period (2008–2018)		
	No. of studies	No. of isolates tested	Proportion of susceptible isolates (% [range])	No. of studies	No. of isolates tested	Proportion of susceptible isolates (% [range])
Escherichia coli (36–38, 43, 44, 61, 67, 71)						
Group A						
Ampicillin	2	67	6.0 (4.2–7.0)	3	26	23.1 (14.3–40.0)
Gentamicin	4	89	74.2 (0.0–93.0)	3	26	76.9 (28.6–100)
Group B						
Ceftriaxone	0	0		3	26	92.3 (85.7–100)
Ciprofloxacin	1	24	91.7 (91.7)	3	26	76.9 (57.1–80.0)
Imipenem-meropenem	1	24	100 (100)	2	21	100 (100)
Trimethoprim-sulfamethoxazole	4	89	5.6 (0.0–12.5)	3	26	34.6 (0–57.1)
Typhoidal *Salmonella* (35, 37, 43, 44, 49, 68, 71, 73)						
Group A						
Ampicillin	4	99	100 (100)	1	45	48.9 (48.9)
Group B						
Chloramphenicol	6	126	99.2 (95.8–100)	1	45	51.1 (51.1)
Ciprofloxacin	1	59	100 (100)	2	66	95.5 (90.5–100)
Trimethoprim-sulfamethoxazole	4	64	81.3 (50.0–100)	1	45	51.1 (51.1)
Ampicillin, chloramphenicol, and trimethoprim-sulfamethoxazole^*b*^	0	0		2	66	56.1 (42.2–85.7)
Nontyphoidal *Salmonella* (33, 35, 43, 44, 61, 67, 73)						
Group A						
Ampicillin	6	331	22.1 (0.0–100)	1	10	10.0 (10.0)
Group B						
Chloramphenicol	5	482	77.8 (0.0–100)	1	10	10.0 (10.0)
Ciprofloxacin	0	0		1	10	90.0 (90.0)
Trimethoprim-sulfamethoxazole	6	486	31.3 (0.0–100)	1	10	10.0 (10.0)
Ampicillin, chloramphenicol, and trimethoprim-sulfamethoxazole^*b*^	0	0		0	0	
Staphylococcus aureus (33, 36–38, 43, 61, 67, 68, 71)						
Group A						
Erythromycin	3	21	90.5 (71.4–100)	1	5	100 (100)
Methicillin-oxacillin	4	16	75.0 (0.0–100)	4	29	69.0 (52.9–100)
Penicillin	4	36	2.8 (0.0–6.7)	2	22	4.5 (0.0–20.0)
Trimethoprim-sulfamethoxazole	3	28	71.4 (69.2–100)	3	23	60.9 (0.0–100)
Group B						
Tetracycline	1	1	100 (100)	2	18	66.7 (0.0–70.6)
Vancomycin	2	20	100 (100)	2	22	100 (100)
Streptococcus pneumoniae (32, 37, 40, 41, 43, 44, 61, 67, 68, 71, 73)						
Group A						
Erythromycin	3	192	99.0 (98.4–100)	3	25	96.0 (85.7–100)
Penicillin	5	254	86.6 (63.6–100)	4	28	85.7 (66.7–100)
Trimethoprim-sulfamethoxazole	4	233	16.3 (1.8–100)	4	28	39.3 (16.7–66.7)
Chloramphenicol	5	246	82.1 (74.4–100)	4	28	100 (100)
Group B						
Ceftriaxone	0	0		2	11	100 (100)
Tetracycline	3	192	59.9 (50.4–70)	1	3	66.7 (67.0)

a As defined by the Clinical and Laboratory Standards Institute (95).

b Defines multiple drug resistance in Salmonella enterica.

There were 11 studies with antimicrobial susceptibility results for S. pneumoniae (32, 37, 40, 41, 43, 44, 61, 67, 68, 71, 73). The proportions of isolates susceptible to erythromycin, penicillin, and trimethoprim-sulfamethoxazole for the earlier period versus the contemporary period were 190 (99.0%) of 192 isolates versus 24 (96.0%) of 25 isolates, 220 (86.6%) of 254 isolates versus 24 (85.7%) of 28 isolates, and 38 (16.3%) of 233 isolates versus 11 (39.3%) of 28 isolates, respectively.

Eight studies reported antimicrobial susceptibility results for E. coli (36–38, 43, 44, 61, 67, 71). Among E. coli isolates with ampicillin susceptibility testing, 4 (6.0%) of 67 were susceptible in the earlier period versus 6 (23.1%) of 26 in the contemporary period. Among E. coli isolates with ciprofloxacin susceptibility testing, 22 (91.7%) of 24 were susceptible in the earlier period (36) versus 20 (76.9%) of 26 in the contemporary period (38, 67, 71). Eight studies tested S. aureus isolates for methicillin resistance (33, 36–38, 43, 67, 68, 71). The proportions of S. aureus isolates susceptible to methicillin were 12 (75.0%) of 16 in the earlier period versus 20 (69.0%) of 29 in the contemporary period. A meta-analysis of the proportions of organisms susceptible to all drugs reported regardless of clinical application in the earlier period versus the contemporary period showed 95.9% (95% CI, 89.7 to 99.5%) versus 77.1% (95% CI, 59.1 to 91.3%) for typhoidal *Salmonella*, 64.1% (95% CI, 49.7 to 77.3%) versus 51.4% (95% CI, 21.8 to 80.6%) for NTS, 76.0% (95% CI, 56.0 to 91.6%) versus 81.2% (95% CI, 68.1 to 91.6%) for S. pneumoniae, 44.5% (95% CI, 26.7 to 63.0%) versus 61.9% (95% CI, 46.8 to 76.0%) for E. coli, and 72.2% (95% CI, 60.8 to 85.7%) versus 57.4% (95% CI, 35.5 to 78.0%) for S. aureus.

## DISCUSSION

We show that CO-BSIs continue to play a major role in febrile ED consultations and hospitalizations. S. enterica, S. pneumoniae, and E. coli were the leading pathogens in Africa and Asia, and BSI were more common in adult studies than in pediatric studies. Although our search was global, studies located in Africa and Asia predominated and are the focus of this review. We identified a number of regional differences, including greater proportions of CO-BSI in participants from studies in Africa, compared to studies in other locations. Several lines of evidence demonstrate increasing prevalence of AMR among community-onset pathogens causing bacteremia.

Salmonella enterica was the leading cause of CO-BSI in Africa and Asia, with nontyphoidal serovars playing a major role in African studies and typhoidal serovars being common in both African and Asian studies. We demonstrate that the proportions of typhoidal and nontyphoidal *Salmonella* isolates susceptible to the traditional first-line drugs ampicillin, chloramphenicol, and trimethoprim-sulfamethoxazole have all declined since 2008. While we did not demonstrate a change in the proportions of S. enterica isolates susceptible to fluoroquinolones between the two time periods, and susceptibility to extended-spectrum cephalosporins was rarely reported, outbreaks of *S. enterica* serovar Typhi and NTS disease resistant to many antimicrobial classes are of great concern (19, 20, 75, 76). Progress to improve access to microbiologically safe water and food and improved sanitation are needed to prevent *Salmonella* infections (77). Typhoid conjugate vaccines represent a new tool for typhoid fever control in areas in which the disease is endemic (78, 79).

Consistent with incidence data (80), we show that S. pneumoniae remains a leading cause of CO-BSI. S. pneumoniae was as common in pediatric studies as in adult studies in Africa and Asia. We identified no statistically significant changes in S. pneumoniae antimicrobial susceptibility between the time periods. Encouragingly, pneumococcal conjugate vaccine was introduced in 142 (73.2%) of 194 World Health Organization member states by 2018, and levels of pneumococcal conjugate vaccine coverage in these states have increased (81, 82).

E. coli was the third most frequently isolated cause of CO-BSI and was more common in adult studies than in pediatric studies. While we observed increases in the proportions of E. coli susceptible to penicillin and trimethoprim-sulfamethoxazole from the earlier period to the contemporary period, the prevalence of fluoroquinolone susceptibility appears to be declining. Of concern, resistance to extended-spectrum cephalosporins among a substantial minority of E. coli isolates causing CO-BSI was observed in studies from the contemporary period. Furthermore, vaccines and other measures to prevent community-onset extraintestinal pathogenic E. coli infections remain at an early stage of development (83).

Our review had a number of limitations. First, although we expanded the search to encompass CO-BSI globally, there were still many regions and countries lacking eligible studies. Second, antimicrobial susceptibility data were available only from studies performed in Eastern Africa and South and South-eastern Asia, limiting our ability to make regional comparisons. Also, the total number of isolates undergoing susceptibility testing in studies included in our review was relatively small. Our AMR findings can be complemented by AMR data generated from national laboratory reporting surveillance networks (84) and other sources, such as large, single-center studies showing AMR trends for common organisms (85). Third, using prospective cohort studies as our data source meant that the substantial data from high-income countries with robust and routine local and national CO-BSI surveillance were not included. However, the primary purpose of this review was to provide data for settings that rely on sentinel site studies to understand the local and national epidemiology of CO-BSI. Fourth, our inclusion criteria of only febrile hospitalized patients did not capture the important group of individuals with nonfebrile CO-BSI, including those with sepsis (86, 87). Fifth, antimicrobial susceptibility standards and interpretive criteria change over time, which can contribute to changes in apparent antimicrobial susceptibility interpretations and results. Because we did not have access to raw antimicrobial susceptibility testing data, we were unable to reinterpret results with contemporary criteria. Sixth, some pathogens are likely underestimated due to incomplete identification. We identified incomplete use of mycobacterial blood cultures and malaria and HIV diagnostic testing for febrile inpatients. Pathogens such as Burkholderia pseudomallei may also be missed through limitations of media and identification methods in some locations (88, 89). Finally, while our review is focused on CO-BSI in the context of febrile illness sufficiently severe to present at an ED or admission to a hospital, we recognize that CO-BSI play an important role in the pathogenesis of the separate but overlapping clinical syndrome of sepsis. However, others have examined the infectious etiology of sepsis, and our study was designed to inform the management of febrile patients in the era of declining malaria incidence.

Our findings support the value of surveillance and high-quality research on CO-BSI to inform empirical treatment strategies, to help set priority pathogens to inform disease control measures, and to highlight the concerning growth of AMR among serious infections cause by community-onset pathogens. While the overall proportion of febrile participants with BSI declined in studies performed after 2008, compared to prior years, we confirm that CO-BSI remain a major cause of febrile presentation for emergency care or hospitalization and that AMR is a growing problem among CO-BSI. Our findings underscore the importance of both non-vaccine-based and vaccine-based control of community-onset pathogens such as *S. enterica* serovar Typhi and S. pneumoniae and highlight the prevention and control gap for E. coli acquired outside the health care system. The control of antimicrobial misuse in people, animals, and the environment is likely essential to slow the emergence of AMR in CO-BSI pathogens (90). Ongoing surveillance and further sentinel site studies remain invaluable for informing empirical management of severe febrile illnesses and bacteremia, as well as guiding strategies to control AMR.

## MATERIALS AND METHODS

### Search strategy and selection criteria.

We performed a systematic review by searching PubMed, Web of Science, and Scopus on 19 September 2018 to identify studies of CO-BSI. The search included key words of fever, bacteremia, septicemia, epidemiology, incidence, and prevalence, as well as spelling alternatives and related terms (see Text S1 in the supplemental material). No restrictions were placed on study setting (e.g., inpatient versus outpatient setting), language, country, or date. Selection criteria were set prior to the initial database searches.

Only prospective studies with consecutive series of febrile patients, with fever as the primary criterion for obtaining blood culture and aerobic or mycobacterial blood culture as the reference standard diagnostic test, were included. If a study enrolled a broader group of afebrile patients (e.g., suspicion of meningitis without fever), it was included only if the initial enrollment criterion was fever. However, we placed no restriction on how fever was defined. We defined CO-BSI as a pathogen-positive blood culture drawn from a febrile patient within 48 h after admission (16). Studies that did not present sufficient detail for calculation of the prevalence of isolates from blood cultures or that reported a single pathogen (e.g., only Streptococcus pneumoniae) as a cause of febrile illness, without describing other causes, were excluded.

Search results from each database were imported into Endnote X8 (Clarivate Analytics, Boston, MA). We also included all references from the bibliographies of the two previous systematic reviews on CO-BSI in Africa and Asia (25, 26). Endnote was used to remove duplicates, and a final deduplicated data set was uploaded to an online systematic review tool for abstract and full-text screening (91).

Two authors screened titles and abstracts for inclusion. Studies included by either author were moved forward to full-text review. All full-text articles were then independently screened in parallel by two authors. Discrepancies were resolved through discussion and, if necessary, by a separate author. Subsequent processes were also performed in this manner.

After the initial full-text review, studies were restricted to hospitalized patients, in keeping with the earlier reviews. Studies in an ED were deemed relevant and were included, on the basis that ED patients are part of a pathway to hospitalization. The full-text versions of included articles were rescreened in parallel, to exclude studies performed in an outpatient setting. Lastly, bibliographies of the final included articles were screened for additional relevant studies. We used the preferred reporting items for systematic reviews and meta-analyses (PRISMA) to record the search process (92). Descriptive study characteristics and quantitative data were abstracted in a shared Google (Mountain View, CA) spreadsheet document.

### Data analysis.

Study quality was assessed by using criteria that aligned with the aims of this review. Our goal was to create an assessment tool that evaluated the quality of a study’s blood culture results and its recruitment procedures. We included two measures important to the growth of microorganisms in blood culture, namely, volume adequacy of culture bottles and the proportion of bottles reported as contaminated. Other questions assessed the possibility of selection bias, such as the study being performed in an ED setting where all patients may not be hospitalized. Quality assessment was performed by two authors in parallel, and discrepancies of the overall score (high, moderate, or low) were resolved through discussion.

Data on individual isolates were compiled and aggregated in Excel (Microsoft, Redmond, WA). If a study did not report the number of BSI, we made the assumption that the total number of pathogens isolated equaled the number participants with BSI and *vice versa*. No other data were imputed to account for missing values. For Salmonella enterica, when a serovar was provided, we grouped serovars Typhi, Paratyphi A, Paratyphi B, and Paratyphi C as typhoidal *Salmonella* and all others as NTS (93).

Isolates were stratified in two ways. First, studies were stratified by age group using the inclusion or median age. Studies with participants ≤15 years of age were defined as pediatric studies. Studies of populations of mixed ages or with median ages of >15 years were defined as adult studies. Comparisons between pediatric and adult studies were made using a two-sample test of proportions in R v3.5.1, with the prop.test function. Second, we stratified by United Nations region (94), describing the prevalence in studies performed in Africa and Asia and in studies performed outside those two regions using MapChart (https://mapchart.net/detworld.html). Additionally, we analyzed the association of HIV or malaria coinfection with BSI overall and also with specific causes of bacteremia. The significance of the associations was determined by the χ^2^ test or Fisher’s exact test.

As a secondary analysis, we abstracted data on antimicrobial susceptibility, when available. We defined an isolate as susceptible when a study reported its susceptibility to specific antimicrobial drugs as susceptible or intermediate and resistant when the study reported the isolate as resistant. We accepted the original study’s classification of isolate antimicrobial susceptibility and did not attempt to access and to reinterpret zone sizes or MIC values based on contemporary interpretive criteria.

Contemporary isolates were defined as those collected in 2008 through 2018. We compared the prevalence of susceptibility between contemporary isolates and earlier isolates (collected prior to 2008) for major drug-organism combinations. We used Clinical and Laboratory Standards Institute suggested antimicrobial agent groups A and B as a guide for reporting specific clinically relevant drugs according to organism group (95). To evaluate trends in overall susceptibility to all drugs that were tested, regardless of clinical importance or application, we also performed a random-effects meta-analysis of proportions of susceptibility over the two time periods, using MetaXL (EpiGear International Pty Ltd.). The study protocol was registered with PROSPERO (accession no. CRD42018109388). Because this was a study involving secondary analysis of published data, institutional review board approval was not required.

## Supplementary Material

Supplemental file 1
